# Ulotaront: review of preliminary evidence for the efficacy and safety of a TAAR1 agonist in schizophrenia

**DOI:** 10.1007/s00406-023-01580-3

**Published:** 2023-05-10

**Authors:** Eric D. Achtyes, Seth C. Hopkins, Nina Dedic, Heather Dworak, Courtney Zeni, Kenneth Koblan

**Affiliations:** 1 WMU Homer Stryker M.D. School of Medicine, Kalamazoo, MI USA; 2 Sunovion Pharmaceuticals Inc., Marlborough, MA USA

**Keywords:** Trace amine-associated receptor 1, Serotonin 5-HT1A, Schizophrenia

## Abstract

Ulotaront is a trace amine-associated receptor 1 (TAAR1) agonist in Phase 3 clinical development for the treatment of schizophrenia. Ulotaront was discovered through a unique, target-agnostic approach optimized to identify drug candidates lacking D2 and 5-HT2A receptor antagonism, while demonstrating an antipsychotic-like phenotypic profile in vivo*.* The mechanism of action (MOA) of ulotaront is thought to be mediated by agonism at TAAR1 and serotonin 5-HT1A receptors. Ulotaront has completed two Phase 2 trials (4-week acute study and 26-week open-label extension) which led to Breakthrough Therapy Designation from the US Food and Drug Administration for the treatment of schizophrenia. In the double-blind, placebo-controlled, acute study, ulotaront was associated with significant (*p* < 0.001) improvement in Positive and Negative Syndrome Scale (PANSS) total score (effect size [ES]: 0.45), with improvements vs. placebo also observed across secondary endpoints. Post-hoc analyses of the acute trial revealed additional evidence to support the effect of ulotaront on negative symptoms. In the 4-week study, ulotaront was well-tolerated, with an incidence of adverse events (AEs) numerically lower compared to placebo (45.8% vs. 50.4%; with a number needed to harm [NNH] for individual ulotaront AEs all > 40). The open-label extension demonstrated further improvement across schizophrenia symptoms and confirmed the tolerability of ulotaront, with a 6-month completion rate of 67%. Based on current data, ulotaront shows potential to be a first-in-class TAAR1 agonist for the treatment of schizophrenia with a safety and efficacy profile distinct from current antipsychotics.

## Introduction: unmet needs in schizophrenia

Schizophrenia is a chronic, multifaceted disorder that consists of symptoms largely grouped into positive, negative, and cognitive domains [1]. Onset typically occurs in late adolescence or early adulthood, and for most patients is characterized by a high degree of chronicity characterized by multiple relapses and remissions (typically partial). [2–4]. While lifetime prevalence of schizophrenia is approximately 1%, the early onset, illness chronicity, and degree of associated functional impairment result in the disorder being ranked among the leading causes of disability and economic burden worldwide [5, 6].

For the past 70 years, the treatment of schizophrenia has relied on antipsychotic drugs whose primary mechanism of action (MOA) is via blockade of the dopamine, type 2 (D2) receptor [7, 8]. Second-generation (atypical) antipsychotics (SGAs), introduced almost 30 years after the first-generation antipsychotics (FGAs), also act primarily via a dopamine inhibiting MOA; however, SGAs are also characterized by antagonist activity at the serotonin 5-HT2A receptor [9]. Except for clozapine, SGAs are not associated with significant improvement in efficacy compared to first-generation antipsychotics [10]. Treatment with high potency SGAs and FGAs can be associated with an increased prevalence of movement disorder symptoms and hyperprolactinemia. Additionally, weight gain and adverse metabolic effects with many SGAs and low potency FGAs may contribute to the increased cardiovascular morbidity and mortality [11, 12].

Among individuals with schizophrenia, it is estimated that approximately one-third are non-responders to currently available antipsychotics, while the majority achieve only partial symptom control [13]. In addition to failing to control positive symptoms in some treatment-resistant patients, relapse rates remain high among patients with schizophrenia taking antipsychotics [1]. Due to the high risk of relapse in this patient population, treatment guidelines consistently recommend maintenance therapy with antipsychotics [14]. Unfortunately, for a large proportion of patients, the benefit-risk profile is unfavorable for both first- and second-generation antipsychotics, resulting in discontinuation rates at one year of greater than 50% [15], though use of long-acting injectable antipsychotics may improve long-term adherence [16]. Each relapse makes remission more difficult to achieve, and failure to prevent relapse leads to consequences in both patient health and disease trajectory.

Negative and cognitive symptoms are the least responsive to antipsychotic treatment, while contributing, in large measure, to the comorbidity, poor health-related quality of life, and chronic disability associated with schizophrenia [1, 17–23]. These two symptom domains are clinically present in the majority of patients with schizophrenia. In an analysis of 20 randomized clinical trials in schizophrenia [24], 62% of patients presented with “prominent” negative symptoms (multiple negative symptoms that were moderate-or-greater in severity); in 20–40% of patients, negative symptoms become a persistent feature of their illness [25]. Clinically significant cognitive impairment exhibits a prevalence in schizophrenia that is comparable to, and as persistent as, negative symptoms, occurring in more than 50% of patients [26, 27]. Reasons for the lack of efficacy of first- and second-generation antipsychotics in effectively treating negative symptoms and cognitive impairment in schizophrenia is uncertain. It has been hypothesized that reduced dopaminergic function in the frontal cortex (and possibly alterations in serotonergic and glutamatergic neurotransmission) may be the neural substrates underlying these two symptom domains [28]. Thus, currently available antipsychotics, acting via antagonism or partial agonism at the D2 receptor, would not be expected to enhance dopaminergic function in the frontal cortex, and in fact, negative symptoms specifically are often observed to be a medication side effect [29].

A substantial investment of research and development resources has been made over the past decade in an attempt to develop drugs with novel, non-D2 MOAs for the treatment of schizophrenia, with particular focus placed on negative and cognitive symptom domains, in addition to an improved tolerability and safety profile [30]. To date, these research efforts have met with little success. However, adjunctive treatments that target specific domains, such as the GlyT1 inhibitor iclepertin, have shown potential [31].

The aim of the current review article is to present an overview of the preclinical and clinical data to date for ulotaront, a novel trace amine-associated receptor 1 (TAAR1) agonist in development for the treatment of schizophrenia, with potential to be one of the first drugs with a non-D2 MOA.

## Discovery and characterization of ulotaront

Ulotaront is a trace amine-associated receptor 1 (TAAR1) agonist with additional agonism at 5-HT1A receptors currently in Phase III clinical development for the treatment of schizophrenia. Ulotaront recently reached recommended status for its proposed International Nonproprietary Name (INN), joining TAAR1 partial agonist ralmitaront in the “-taront” class of medications. Ulotaront is the first agent in this novel class of compounds to demonstrate clinical efficacy in a randomized, double-blind, placebo-controlled Phase 2 trial in patients with acute schizophrenia, thus representing a potential “new treatment paradigm” [1]. Based on Phase 2 data, ulotaront was granted Breakthrough Therapy Designation from the U.S. Food and Drug Administration for the treatment of schizophrenia.

**Fig. 1:**
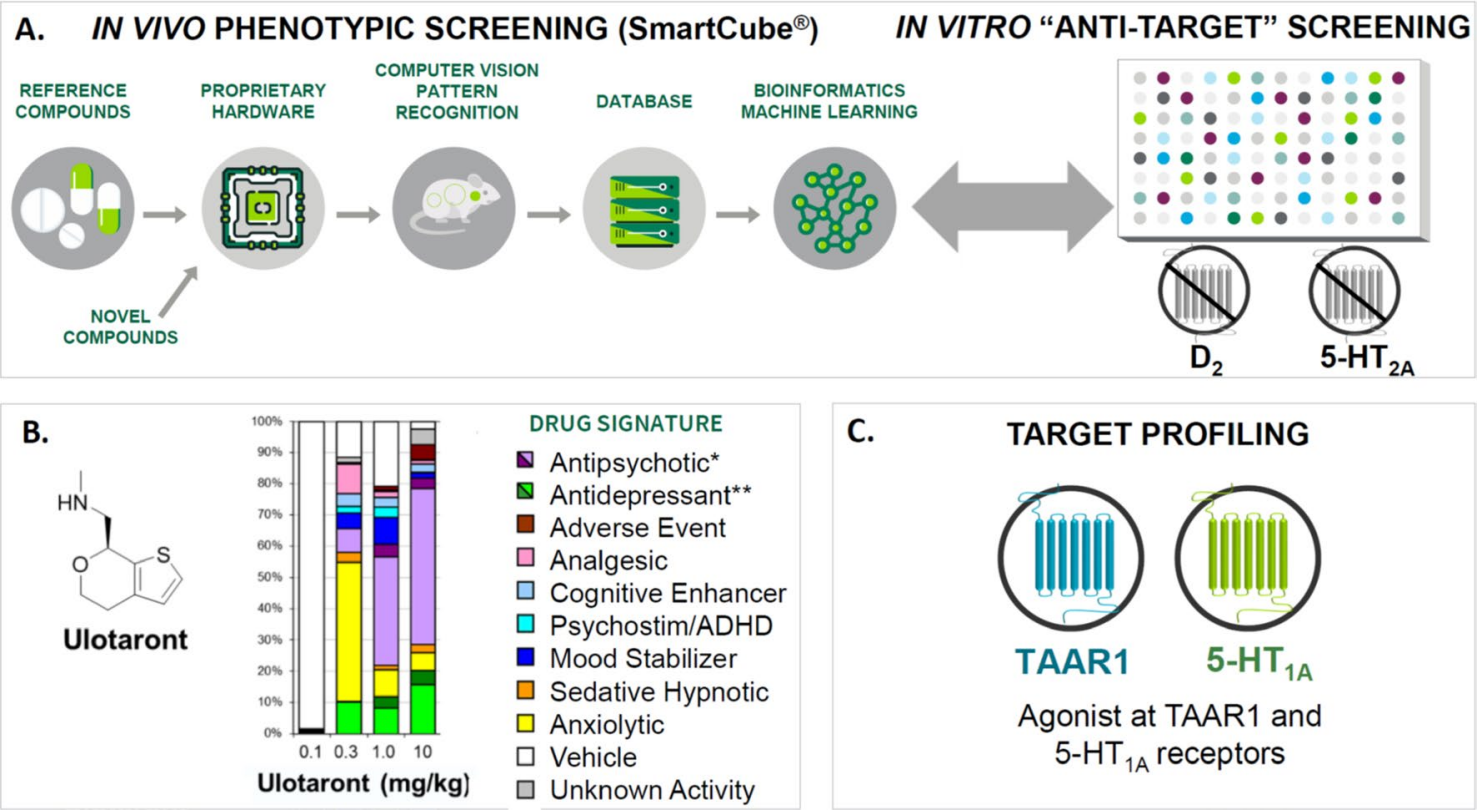
Discovery and characterization of ulotaront. **A** Ulotaront discovery. Ulotaront was discovered through a unique, target-agnostic approach designed to identify drug candidates that lack D_2_ and 5-HT_2A_ receptor antagonism but retain an antipsychotic-like behavioral profile in vivo*.* **B** Ulotaront mouse SmartCube^®^ profile. Ulotaront demonstrated a predominantly antipsychotic-like behavioral profile (drug signature), with some secondary anxiolytic and antidepressant-like activity. **C** In vitro target profiling of ulotaront (SEP-363856). Additional in vitro and in vivo pharmacological studies showed that agonism at TAAR1 and 5-HT_1A_ receptors contribute to the efficacy of ulotaront [32, 35]. *D2* dopamine D2 receptor, *5-HT1A and 5-HT2A* serotonin 1A and 2A receptor subtypes, *ADHD* attention deficit disorder with hyperactivity. *Antipsychotic (purple) and high-dose antipsychotic (dark purple); **antidepressant (green) and high-dose antidepressant (dark green)

Ulotaront was discovered through a unique, target-agnostic approach designed to identify drug candidates that lack D2 and 5-HT2A receptor antagonism yet retain an antipsychotic-like behavioral profile when evaluated in rodents in vivo [32]. In brief, through iterations of the general screening process outlined in Fig. 1A followed by secondary assays, ulotaront was selected for further development [32]. In the in vivo phenotypic screening platform, SmartCube^®^, ulotaront demonstrated a predominantly antipsychotic-like behavioral profile, with some secondary anxiolytic-, and antidepressant-like activity (Fig. 1B) [32]. Ulotaront is the first, and currently only, compound discovered through this target-agnostic approach to advance to proof-of-concept clinical studies [33, 34].

## Preclinical pharmacology

Functional profiling in vitro showed that ulotaront is a full agonist (E_max_ = 101%) at TAAR1 (EC_50_ = 0.14 µM). Ulotaront also exhibits binding to serotonin 5-HT_1A_ receptors (Ki = 0.28 µM) where it acts as an agonist (EC_50_ = 2.3 µM; E_max_ = 75%). Subsequent mechanistic studies demonstrated that these receptor activities contribute to the effects of ulotaront in vivo (summarized below). In addition, ulotaront has affinity for serotonin 5-HT1D (Ki = 1.13 µM), 5-HT1B (Ki = 1.9 µM) and 5-HT_7_ (Ki = 0.03 µM) receptors, although only weak agonism was reported for 5-HT1B (EC_50_ = 15.6 µM; E_max_ = 22%) and 5-HT7 (EC_50_ = 6.7 µM; E_max_ = 41%) [32]. No appreciable binding, functional activity and/or in vivo receptor occupancy was seen at dopamine D_2_ or serotonin 5-HT_2A_ receptors. For more details relating to the in vitro receptor profiling, we refer the reader to the original research article [32].

TAAR1 is a G-protein coupled receptor (GPRC) that is widely expressed in the rodent brain, albeit at very low levels. In rodents, receptor expression has been reported in monoaminergic nuclei including the ventral tegmental area (VTA), substantial nigra (SN), and dorsal raphe nucleus (DRN) as well as limbic brain regions (e.g., amygdala, hippocampus), basal ganglia, and the prefrontal cortex [36, 37]. Thus, it is not surprising that TAAR1 has been shown to affect dopaminergic, serotonergic and glutamatergic signaling and consequently modulate aspects of reward-processing, cognition and mood relevant to schizophrenia and other psychiatric disorders [38, 39]. Due to its low expression levels and lack of suitable, commercially-available tools such as antibodies for TAAR1, the synaptic and cellular localization of TAAR1 remains largely unexplored. Intracellular receptor localization has been reported, with evidence for plasma membrane expression following ligand-induced heterodimerization with other GPCRs [35, 37, 40–46].

TAAR1 agonists are broadly active in preclinical models/assays and have demonstrated antipsychotic, anti-addictive, pro-cognitive, antidepressant-like, and wake-promoting effects [35, 38, 39]. Ulotaront has been extensively studied in rodent models relevant to schizophrenia, supported by its antipsychotic-like profile in the i*n vivo* phenotypic screening platform SmartCube^®^ [32]. Efficacy has been reported in several additional models/assay, including stimulant-induced deficits/alterations in locomotor activity, prepulse inhibition, social interaction and cognition (Table 1). Importantly, some of the in vivo effects (including antipsychotic-like activity) were absent in TAAR1-knock out mice confirming the contribution of TAAR1 to ulotaront’s mechanism of action [35]. In addition, 5-HT_1A_ receptors were shown to partially contribute towards the effects of ulotaront in the mouse PCP-induced hyperactivity assay. Although evidence exists for the therapeutic effects of 5-HT_1A_ agonists in depression and anxiety-related disorders [47], the potential for efficacy of 5-HT_1A_ agonists in schizophrenia, particularly in combination with TAAR1 agonism, remains to be explored.

**Table 1:** Pharmacologic effects of ulotaront in preclinical models

*Effects in rodent models of schizophrenia*
Antipsychotic-like behavioral profile in the SmartCube^®^ platform (mouse) [32]
Reduction of PCP-induced hyperactivity (mouse/rat) [32, 58]
No effect on amphetamine-induced hyperactivity (rat) [58]
Reversal of sub-chronic PCP-induced social interaction deficits (rat) [32]
Increase in prepulse inhibition (PPI; mouse) [32]
Attenuation of MK-801-induced deficits in PPI and hyperactivity (mouse) [35]
Reversal of sub-chronic PCP-induced deficits in object recognition memory (rat) [58]
Reversal of the sub-chronic ketamine-induced increase in striatal dopamine synthesis capacity (mouse) [53]
Potentiation of olanzapine effects on apomorphine-induced climbing and MK-801-induced hyperactivity (mouse) [59]
*Effects in other rodent models*
Modest reduction of immobility in the forced swim test (FST) and possible potentiation of duloxetine effects in the FST and TST (mouse) [32, 60]
Reduction of olanzapine-induced body weight gain (mouse) [59]
Reduction of cocaine cue-reinstated responding (rat) [61]

The ability of TAAR1 to modulate dopaminergic circuits has attracted considerable interest in the context of schizophrenia and psychosis in general. VTA neuronal firing and electrically evoked dopamine release are reduced by TAAR1 full agonists, whereas generally opposite effects are seen with the TAAR1 antagonists such as *N*-(3-Ethoxy-phenyl)-4-pyrrolidin-1-yl-3-trifluoromethyl-benzamide (EPPTB) as well as in TAAR1-KO mice [48–52]. Interestingly, the inhibitory effects on dopaminergic neurotransmission appear to be most pronounced under hyperdopaminergic conditions. This is supported by recent findings, showing that ulotaront reduces the ketamine- induced increase in striatal dopamine synthesis capacity without producing an effect in naïve mice [53]. Elevated dopamine synthesis capacity has repeatedly been reported in schizophrenia patients and is not targeted by current antipsychotic treatments [54–57]. Whether ulotaront’s effects on dopamine synthesis capacity are mediated through direct action on midbrain dopaminergic neurons, or via upstream modulation of glutamatergic circuits, remains to be determined.

In contrast to some of the currently available antipsychotic drugs, and consistent with the lack of D2 receptor activity, neither selective TAAR1 agonists nor ulotaront induce catalepsy in rodents [32, 50]. Thus, TAAR1 agonists are unlikely to cause D2 antagonist-mediated extrapyramidal side effects (EPS, movement disorders), which constitute well-known side effect of the current class of antipsychotic drugs. Interestingly, TAAR1 agonists, including ulotaront, have also been reported to potentiate the antipsychotic properties of olanzapine and/or risperidone, highlighting their potential as adjunctive treatments to current antipsychotics [50, 59]. In addition, an increasing number of studies are implicating TAAR1 in the potential regulation of metabolic function and food reward behavior [38, 48, 62]. This could be of significant relevance considering that obesity, hyperglycemia, insulin resistance and dyslipidemia constitute major side effects of antipsychotic medication [63]. In contrast, TAAR1 agonists have been shown to decrease body weight in naïve rodents, prevent olanzapine-induced weight gain in rats, reduce food intake and excess body weight in diet-induced obese mice and attenuate binge-like eating in rats [43, 50, 58, 64]. Additional studies in mouse models of type 2 diabetes mellitus reported improved glucose tolerance and insulin sensitivity, as well as reduced plasma and liver triglyceride levels [42]. The underlying mechanisms may include TAAR1-mediated peripheral effects on glucose homeostasis and gastric emptying, and/or direct modulation of homeostatic and hedonic neurocircuits regulating energy balance. Thus, the current preclinical evidence suggests that TAAR1 agonists hold promise to improve several symptom domains of schizophrenia without causing motor impairments and metabolic dysregulation. In fact, the potential beneficial metabolic effects suggest that TAAR1 agonists may improve comorbid metabolic dysfunction in schizophrenia patients.

## Lack of abuse liability in rodent models

A series of studies conducted in rodent models predictive of abuse potential in humans indicate that ulotaront is not likely to pose a risk of recreational abuse in humans [61]. Notably, single doses of ulotaront were associated with reductions in cocaine-primed induced reinstatement, and dose-dependently reduced cue-reinstated responding [61]. This is consistent with the effects of TAAR1 selective agonists which have been shown to inhibit the rewarding and reinforcing effects of drugs of abuse and drug-abuse related behaviors (reviewed in detail by Gainetdinov et al.; Pei et al.; Liu and Li) [38, 44, 65]. The mechanism is not fully elucidated but likely has its cellular and molecular basis in the attenuation of dopaminergic hyperactivity (i.e., accumulation of DA) induced by drugs of abuse [65].

## Ulotaront: pharmacokinetics profile

The pharmacokinetic (PK) profile of ulotaront in preclinical species and humans has been well-characterized [66, 67]. Ulotaront is a small molecule with high solubility, high permeability, and low plasma protein binding in rodents and humans (unbound fraction, > 78%).

The ability of ulotaront to penetrate the blood–brain barrier has been demonstrated in mice and rats. Following single oral or intraperitoneal administration (10 mg/kg), ulotaront was rapidly absorbed and distributed to the brain with maximum concentrations reached within 30 min post dose in. The brain to plasma ratios (Cmax and AUC) were ≥ 4 and ≥ 2 in mice and rats, respectively [66].

In humans, ulotaront is well-absorbed after oral ingestion with a median time to maximum concentration (Tmax [90%-CI]) of 2.8 [1.0, 6.2] hours and a median effective terminal half-life (t_*½*_) of 7 [4.4, 11.4] hours. Daily dosing to steady state results in an accumulation ratio of 1.1, consistent with a once-daily dosing regimen. The PK profile of ulotaront exhibits a linear relationship between dose and plasma concentration across the presumed therapeutic dose range of 25–100 mg/day. However, the dose- and concentration–response (PK/PD) relationships for ulotaront in the acute (or maintenance) treatment of schizophrenia have not been established.

The metabolic and excretory pathways for ulotaront disposition have been well-characterized. Greater than 92% of ulotaront is excreted in urine as either parent drug (15%) or metabolites. A single major, inactive metabolite has been identified. There are no known clinically meaningful drug-drug interactions involving ulotaront or its metabolites against CYP enzymes or transporters.

## Ulotaront: preliminary evidence for short-term efficacy

Ulotaront is currently being evaluated in a series of Phase 3 clinical trials designed to evaluate its short-term efficacy in the treatment of schizophrenia as well as its long-term effectiveness, safety, tolerability, and effect on measures of function and quality of life (Table 2). The dose selection for evaluation in schizophrenia patients was guided by an initial Phase 1 study in healthy male volunteers demonstrating robust rapid eye movement (REM) sleep-suppressing effects of ulotaront at 50 mg [68]**.**

**Table 2:** Summary of ulotaront clinical development program in schizophrenia (the SEP361-201 and SEP361-202 studies have been completed)

	SEP361-201 (acute study)	SEP361-202 (extension study)	SEP361-301 (acute study)	SEP361-302 (acute study)	SEP361-303 (extension study)	SEP361-304 (long-term safety study)	SEP363-856 (switch study)
Clinicaltrials.gov identifier	NCT02969382	NCT02970929	NCT04072354	NCT04092686	NCT04109950	NCT04115319	NCT05628103
Setting	Inpatient	Outpatient	Inpatient	Inpatient	Outpatient	Outpatient	Outpatient
Study duration	4 weeks	26 weeks	6 weeks	6 weeks	52 weeks	52 weeks	Up to 12 weeks
Sample size	245 (enrolled)	157 (enrolled)	525	462	555	300	~ 120
Age	18–40 years	18–40 years	13–65 years	18–65 years	18–65 years	18–65 years	18–65 years
Population	Acutely psychotic	201-Extension	Acutely psychotic	Acutely psychotic	301 & 302-Extension	Stable patients	Stable patients*
Dosing type	Flexible	Flexible	Fixed	Fixed	Flexible	Flexible	Flexible
Ulotaront dosing	50 mg; 75 mg	25–75 mg	50 mg; 75 mg	50 mg; 100 mg	25–100 mg	50–100 mg	50–100 mg
Comparators	Placebo	None	Placebo	Placebo	None	Quetiapine XR 400–800 mg	None

*Candidate for switching from current antipsychotic medication due to safety or tolerability concerns and/or insufficient efficacy

To date, the short-term efficacy of ulotaront in the treatment of schizophrenia has been evaluated in a Phase 2, multinational, 4-week, randomized, double-blind, parallel-group study of flexibly-dosed ulotaront (50–75 mg/day; n = 120) versus placebo (n = 125) in acutely psychotic adult inpatients with a DSM-5 diagnosis of schizophrenia [69]. The treatment sample was comprised of 64% males, mean age 30 years, with a mean PANSS total score at baseline of 101 (PANSS-positive and -negative subscale scores: 26 and 25, respectively), and a Brief Negative Symptom Scale (BNSS) total score of 37.

**Fig. 2:**
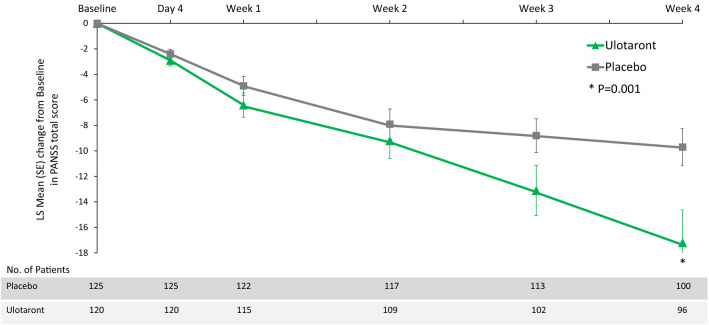
Significant improvement in PANSS total score during 4 weeks of treatment with ulotaront (50–75 mg/day) [69]

Significant improvement in the PANSS total score was observed at Week 4 (the primary efficacy endpoint; effect size, 0.45; Fig. 2), with separation from placebo observed as early as Week 3.

Statistically significant efficacy was also observed at Week 4 across all secondary efficacy measures (Table 3). Effect sizes were generally in the moderate range.

**Table 3:** Baseline to Week 4 change in efficacy measures (MMRM analysis) [69]

Efficacy measure	Ulotaront, 50/75 mg (n = 120)	Placebo (n = 125)	LS mean difference (95%-CI)	Effect size
	LS mean (SE)	LS mean (SE)		
PANSS total score	− 17.2 (1.7)	− 9.7 (1.6)	− 7.5 (− 11.9, − 3.0)	0.45
CGI-S score	− 1.0 (0.1)	− 0.5 (0.1)	− 0.5 (− 0.7, − 0.2)	0.52
PANSS positive subscale score	− 5.5 (0.5)	− 3.9 (0.5)	− 1.7 (− 3.1, − 0.3)	0.32
PANSS negative subscale score	− 3.1 (0.4)	− 1.3 (0.4)	− 1.5 (− 2.6, − 0.4)	0.37
BNSS total score	− 7.1 (1.0)	− 2.7 (0.9)	− 4.3 (− 6.8, − 1.8)	0.48
MADRS total score	− 3.3 (0.6)	− 1.6 (0.6)	− 1.8 (− 3.2, − 0.3)	0.32

*MMRM* Mixed model for repeated measures, *LS* least squares, *SE* standard error, *PANSS* Positive and Negative Syndrome Scale, *CGI-S* Clinical Global Impression, Severity, *BNSS* Brief Negative Symptom Scale, *MADRS* Montgomery-Asberg Depression Rating Scale

Significance testing was not adjusted for multiplicity on secondary efficacy measures

**Fig. 3:**
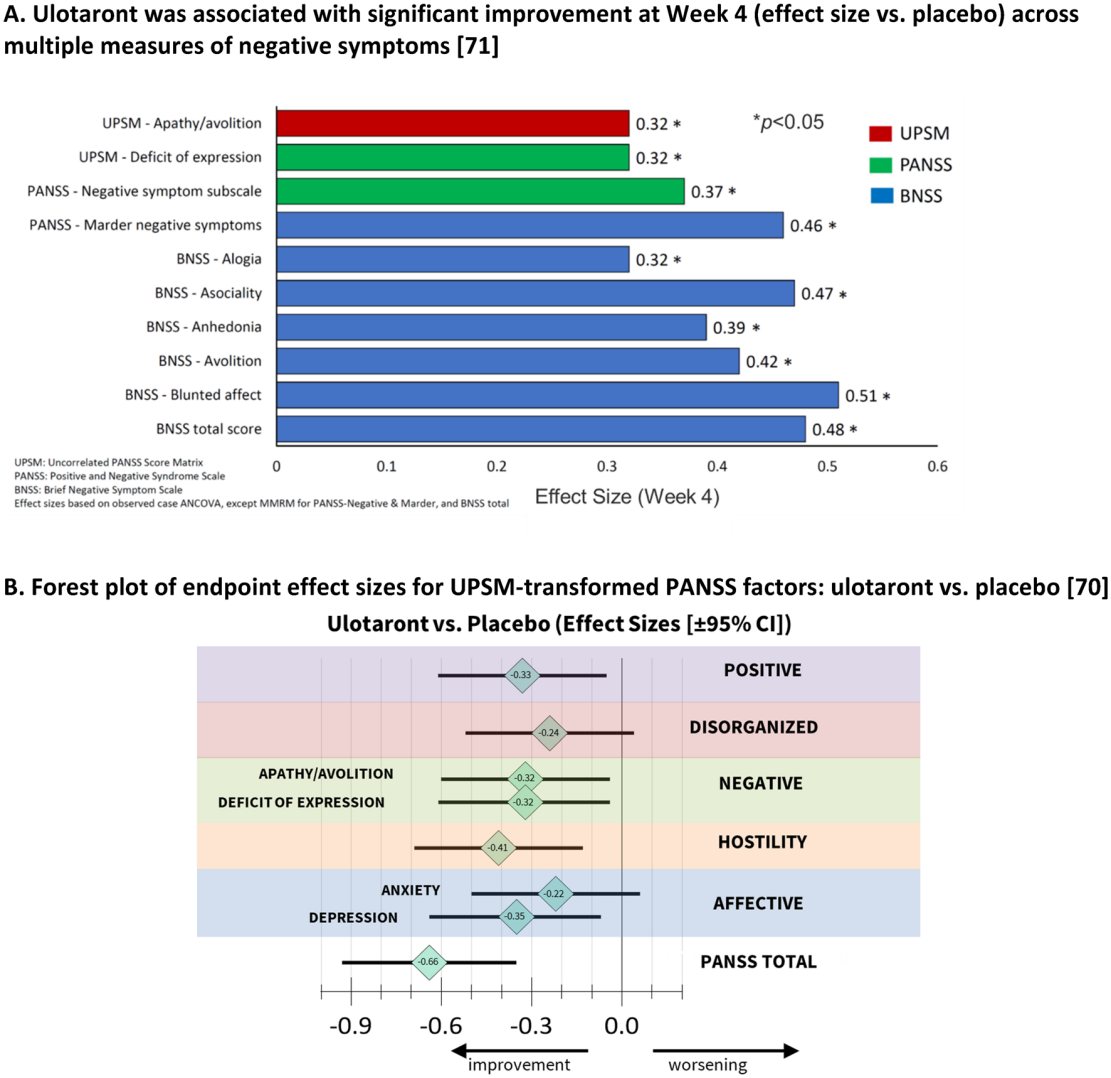
Ulotaront (50–75 mg/day) efficacy in schizophrenia symptom domains in a randomized clinical trial. **A** Ulotaront was associated with significant improvement at Week 4 (effect size vs. placebo) across multiple measures of negative symptoms [70]. **B** Forest plot of endpoint effect sizes for UPSM-transformed PANSS factors: ulotaront vs. placebo [69]

The efficacy of ulotaront on negative symptoms has been examined in more detail in a (pre-specified) analysis of four measures of negative symptoms (Fig. 3A) [70]. The ulotaront vs. placebo effect sizes at Week 4 for the PANSS-Marder negative symptom factor and BNSS total scores were similar (0.46 and 0.48, respectively) while the PANSS-negative subscale score effect size was somewhat lower (0.37).

However, it is well-known that change scores for PANSS-derived negative symptom measures are highly correlated with PANSS measures of positive symptoms. For example, the correlation between the PANSS-Marder positive and negative factor change scores has been shown to be 0.57 [71]. The correlation between PANSS Marder positive symptom factor and the BNSS total score is lower (0.36) but is still significant. These levels of correlation suggest that the improvement in negative symptoms that have been reported for many years for the D2 antipsychotic drugs may be attributed, in no small measure, to PANSS-positive symptom-related effects [71]. To address this cross-correlation issue, a pre-specified analysis of Week 4 improvement in negative symptoms on ulotaront was performed using an uncorrelated PANSS score matrix (UPSM) transformation of the two subdomains of the PANSS-negative symptom factor. The UPSM-transformed factors measure drug effects on PANSS symptom domains in schizophrenia with greater specificity by mathematically reducing the correlated improvements among individual PANSS items, resulting in low (0.04-to-0.10) between-factor correlations for UPSM-PANSS negative subfactors, apathy/avolition and deficit of expression (for a more detailed explanation, see: [71, 72]). The results of this analysis revealed positive effects for the UPSM negative symptom factors, apathy/avolition and deficit of expression (Fig. 3B) remaining over-and-above the expected improvements stemming from correlated effects on other PANSS factors.

To date, only an exploratory cross-study analysis is available based on a post-hoc enrichment strategy that compared endpoint effect sizes for ulotaront vs. placebo and pooled lurasidone vs. placebo on the UPSM-negative symptom factor [73]. In both treatment samples, enrichment was not based on identifying a subgroup with high baseline negative symptom severity. Instead, the enrichment strategy identified a subgroup of patients with low pre-randomization (Screen and Baseline) measurement heterogeneity on the Marder PANSS negative symptom (MPNS) construct. In the subgroup analysis of patients exhibiting high MPNS construct factor validity (with 69% of variance explained versus 37% for the non-enriched subgroup), treatment with ulotaront was associated with a notably larger effect size than lurasidone (0.84 vs. 0.33), suggesting that a prognostic enrichment strategy may be a more-efficient way to establish whether the clinical benefit of ulotaront (versus a D2-class MOA) extends to a specific improvement in negative symptoms [73]. Whether the TAAR1/5-HT1A MOA of ulotaront offers a differential efficacy advantage in the treatment of the negative symptom domain of schizophrenia when compared to D2 antipsychotic compounds awaits the results of ongoing Phase 3 clinical trials (Table 2).

## Ulotaront: preliminary evidence for longer-term effectiveness

Of the 193 patients who completed the 4-week, double-blind, placebo-controlled trial of ulotaront, 157 patients (81.3%) continued into a 26-week open-label (OL) extension study, including 78 patients treated with ulotaront during the double-blind phase, and 79 patients treated with placebo (switching from placebo to ulotaront was accomplished while maintaining the initial study double-blind).

**Fig. 4:**
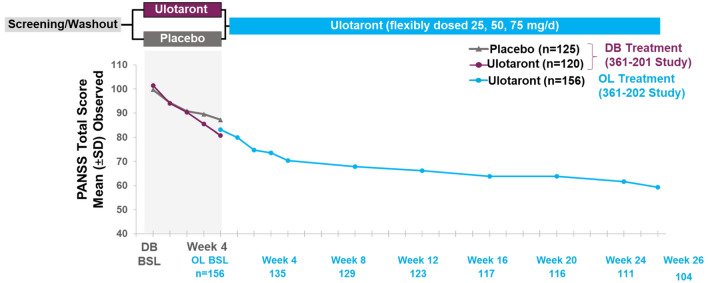
Improvement in PANSS total score during 26 weeks of treatment with ulotaront (50–75 mg/day) [74]. *PANSS* positive and negative syndrome scale, *DB* double blind, *OL* open label, *BL* baseline

Twenty-six weeks of treatment with ulotaront was associated with continued improvement in symptoms of schizophrenia as measured by the PANSS total score (Fig. 4) [74]. In the group of patients who met responder criteria (≥ 30% reduction in PANSS total score) after completing double-blind treatment with ulotaront, the Kaplan–Meier estimate of the probability of relapse at the end of 26 weeks open-label treatment was 0.23.

Continued improvement was also observed on the secondary efficacy measures such as the CGI-S score, the PANSS subscale scores, and the BNSS total score (Table 4). The great majority of patients met responder criteria of ≥ 30% reduction from DB baseline in the PANSS total score. A post-hoc analysis found that a large proportion of patients met stringent ≥ 50% reduction criteria at Week 26 (Table 4).

**Table 4:** Baseline to Week 26 change in efficacy measures (MMRM analysis) [74]

	Mean (SD) change from DB baseline (OC: n = 104)	Mean (SD) change from OL baseline (OC: n = 104)	Mean (SD) change from OL baseline (LOCF: n = 155)
PANSS total score	− 41.8 (14.0)	− 22.6 (15.5)	− 13.8 (21.6)
CGI-S score	− 2.0 (0.8)	− 1.0 (0.9)	− 0.6 (1.2)
PANSS positive subscale score	− 13.5 (4.7)	− 7.3 (5.4)	− 4.5 (7.0)
PANSS negative subscale score	− 8.4 (4.5)	− 5.2 (4.2)	− 3.5 (4.9)
BNSS total score^a^	− 16.8 (12.4)	− 11.3 (9.7)	− 8.0 (11.2)
Reduction from DB baseline in PANSS total score:		≥ 30% reduction	≥ 50% reduction
Responder rates at Week 26		OC: 93.3%	OC: 72.1%
		LOCF: 73.6%	LOCF: 51.2%

*OC* observed case analysis, *LOCF* last observation carried forward analysis, *DB* double-blind, *OL* open-label, *PANSS* Positive and Negative Syndrome Scale, *CGI-S* Clinical Global Impression, Severity, *BNSS* Brief Negative Symptom Scale, *MADRS* Montgomery-Asberg Depression Rating Scale

^a^Sample sizes for BNSS total score: n = 96/100/149

## Ulotaront: preliminary safety and tolerability data

A preliminary safety and tolerability profile of ulotaront can be gleaned from the 4-week double-blind, placebo-controlled Phase 2 study [69], and the 26-week, open-label extension study [70]. As can be seen in Table 5, there were five AE terms that occurred (during the 4-week double-blind study) with an incidence ≥ 2% in the ulotaront group (and greater than placebo); all five AE terms had an incidence < 7% and the number needed to harm (NNH) value for each AE was > 50 for all but one AE (dyspepsia, NNH = 40), indicating that absolute risk increase (i.e., difference in event rate between drug and placebo) is likely to be of minimal clinical concern [75]. The combined incidence of extrapyramidal symptoms (akathisia, restlessness, musculoskeletal or joint stiffness, tremor, and nuchal rigidity) was 3.3% and 3.2% in the ulotaront and placebo groups respectively. Consistent with this favorable tolerability, the proportion of patients reporting any AE was lower on ulotaront compared to placebo (45.8% vs. 50.4%), the rate of AEs rated as “severe” was 5.8%, and the overall discontinuation rate for ulotaront was comparable to placebo (21.7% vs. 20.8%), with discontinuation due to an AE of 8.3% (vs. 6.4% on placebo; Table 5). Serous adverse events (SAEs) occurred in 2 patients treated with ulotaront versus 4 patients receiving placebo.

**Table 5:** Tolerability outcomes in a 4-week double-blind study (AE in the ulotaront group ≥ 2% and greater than placebo); and tolerability and safety in a 26-week open-label extension study (AE ≥ 5%) [69, 74]

Adverse events, 4-week DB	Ulotaront, 50 or 75 mg/day (n = 120)		Placebo (n = 125)
	%	NNH	%
Somnolence	6.7	53	4.8
Agitation	5.0	> 100	4.8
Nausea	5.0	56	3.2
Extrapyramidal symptoms (combined)	3.3	> 100	3.2
Diarrhea	2.5	59	0.8
Dyspepsia	2.5	40	0
Any adverse event	45.8	N/A	50.4
Severe adverse events	5.8	24	1.6
Adverse event leading to discontinuation	8.3	53	6.4

	Ulotaront, 50 or 75 mg/day (n = 156)
Adverse events, 26-week OL extension	%
Schizophrenia	12.2
Headache	11.5
Insomnia	8.3
Anxiety	5.1
Severe adverse events	5.1

OL extension endpoint (week 26)	Ulotaront, 50 or 75 mg/day (n = 156)	
Weight, BMI, laboratory values	Double-blind baseline	Change at Week 26 (OC)
Weight, kg, mean (SD)	75.4 (13.9)	− 0.3 (3.7)
Body mass index, kg/m^2^, mean (SD)	25.1 (3.9)	− 0.1 (1.2)
Total cholesterol, mg/dL, median	174.5	− 2.0
LDL cholesterol, mg/dL, median	101.5	− 9.0
Triglycerides, mg/dL, median	101.0	− 5.0
HbA1c, %, median	5.2	0.0
Prolactin, ng/mL, median		
Female (n = 54)	16.1	− 3.4
Male (n = 102)	11.6	− 2.7

*DB* double-blind, *NNH* number needed to harm, *OL* open-label, *BMI* body mass index, *SD* standard deviation, *LDL* low density lipoprotein, *HbA1c* Glycated hemoglobin, *OC* observed case analysis (sample sizes at week 26 ranged from n = 104 (weight/BMI) to n = 111 (metabolic labs)

The 26-week, open-label extension study [74] provided additional evidence for a favorable benefit-risk ratio of ulotaront, most notably the overall 67% completion rate. As noted in the paper reporting the primary results of this study, [74] this completion rate compares favorably to completion rates at 24 weeks reported in the Clinical Antipsychotic Trials of Intervention Effectiveness (CATIE) study [76], which range from 39% for ziprasidone and quetiapine, to 55% for olanzapine.

On safety parameters, 26 weeks of treatment with ulotaront was associated with safety profile that was different from many of the currently approved antipsychotic medications. Most notably there were no clinically significant changes in median prolactin levels, mean weight, and median metabolic parameters (Table 5). Furthermore, standard movement disorder scales showed no clinically significant changes. For example, changes (mean (SD)) in the Simpson–Angus Scale mean score, Barnes Rating Scale for Drug-Induced Akathisia total score, and the Abnormal Involuntary Movement Scale total score were − 0.0 (0.1), − 0.1 (0.2), and 0.0 (0.1), respectively [74]. Worsening schizophrenia, headaches, insomnia, and anxiety were the only individual adverse events that occurred with an incidence ≥ 5% during 26 weeks of treatment with ulotaront (Table 5).

## Overview of class effect differences between atypical antipsychotics and ulotaront

**Fig. 5:**
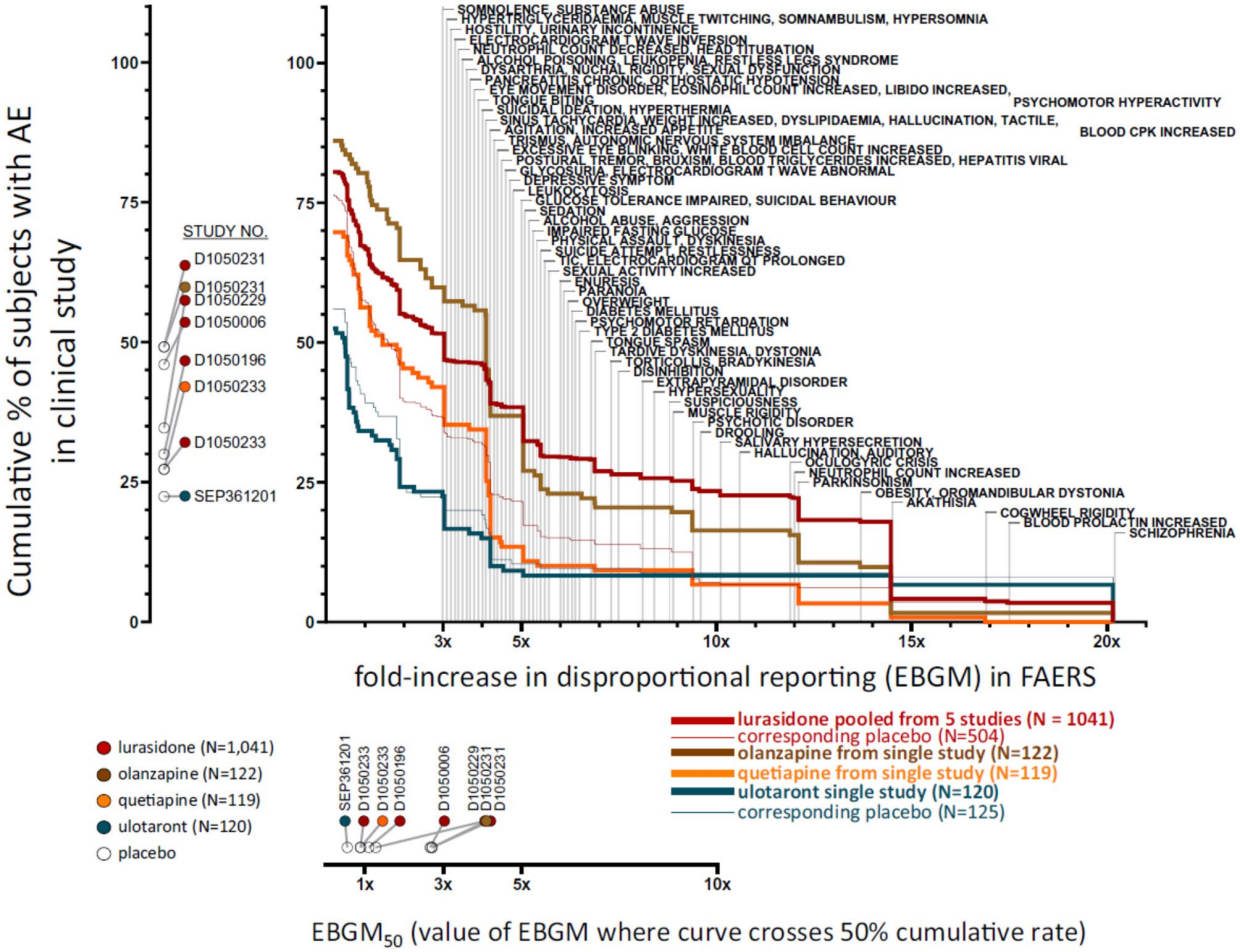
Second generation class-related D2 antipsychotic adverse events based on FAERS data (n = 11 antipsychotics). The x-axis is the fold-increase in disproportional reporting of each AE. The y-axis is the cumulative proportion of patients with class-specific AEs that meet the threefold EBGM threshold [77]. *EBGM* Empirical Bayes Geometric Mean, *FAERS* Food and Drug Administration Adverse Event Reporting System (FAERS) data

Atypical antipsychotic drugs whose MOA is mediated by antagonism at D2/5-HT2A receptors, exhibit a class-specific risk for certain adverse effects (e.g., extrapyramidal symptoms [EPS], cardiometabolic symptoms, hyperprolactinemia). As summarized in detail in recent reports [77] these D2-antipsychotic class-specific preferred terms have been empirically identified, utilizing an Empirical Bayes Geometric Mean (EBGM) disproportionality analysis of Food and Drug Administration Adverse Event Reporting System (FAERS) data, as any preferred term that meets the threefold greater EBGM threshold for drug vs. placebo. The application of the antipsychotic class-effect query has also been applied to historical trials of risperidone, lurasidone, olanzapine, and quetiapine [77, 78] to indicate that approximately half of the adverse events occurring in clinical trials of approved antipsychotic compounds are class effects. To illustrate this result in more detail, Fig. 5 displays the cumulative proportion of patients with schizophrenia having an AE on a second-generation antipsychotic drug at-or-above the threefold disproportional EBGM. As can be seen, there is a notable difference in the cumulative proportion of antipsychotic class-specific AEs for ulotaront when compared to lurasidone, olanzapine, and quetiapine [75].

## Conclusion

Ulotaront is the first TAAR1 agonist that has progressed to Phase 3 clinical trials for the treatment of schizophrenia. Phase 2 clinical data suggest that ulotaront may treat a spectrum of symptoms associated with schizophrenia, including both positive and negative symptoms. In addition, clinical data point to a safety and tolerability profile distinct from the SGA and FGA antipsychotic classes, consistent with the absence of D2 receptor blockade. Though the MOA of ulotaront in the treatment of schizophrenia has not been fully elucidated, a growing body of evidence [32, 35] suggests that the efficacy of ulotaront is mediated through agonism at TAAR1 and serotonin 5-HT1A receptors. Via this novel mechanism, it is thought that ulotaront may provide a distinct risk/benefit profile notably lacking D2 antipsychotic class-related AEs (e.g., EPS, hyperprolactinemia, and adverse weight and metabolic effects). Emerging Phase 3 clinical data from this compound will not only be fundamental to our understanding of ulotaront but may help elucidate the therapeutic utility of TAAR1 agonists for the treatment of schizophrenia and beyond [79–83].
